# Inflammatory Bowel Disease (IBD): Clinical Diagnosis and Treatment

**DOI:** 10.3390/jcm14176237

**Published:** 2025-09-04

**Authors:** Isabel Silva, Moisés Tolentino Bento da Silva

**Affiliations:** 1 Laboratory of Pharmacology and Neurobiology, Center for Drug Discovery and Innovative Medicines (MedInUP)/RISE-Health: Health Research Network, Department of Immuno-Physiology and Pharmacology, School of Medicine and Biomedical Science—University of Porto (ICBAS-UP), 4050-313 Porto, Portugal; issilva@icbas.up.pt; 2 Laboratory of Physiology, Center for Drug Discovery and Innovative Medicines (MedInUP)/RISE-Health: Health Research Network, Department of Immuno-Physiology and Pharmacology, School of Medicine and Biomedical Science—University of Porto (ICBAS-UP), 4050-313 Porto, Portugal

Inflammatory bowel disease (IBD) is a pathological condition that occurs in two different forms: Crohn’s disease (CD) and ulcerative colitis (UC). The etiology is multifactorial and involves any part of the gastrointestinal tract in Crohn’s disease (CD) and significant inflammation limited to the colon in ulcerative colitis (UC). The major symptoms of this incurable disease include severe bowel manifestations such as abdominal pain, diarrhea, constipation, and gastrointestinal discomfort that negatively affect patients’ quality of life [1,2].

In recent years, the search for different types of treatment and diagnostic methods has led to the discovery of new possible biomarkers and other methods of providing a faster and more accurate diagnosis of inflammatory bowel disease [3]. Regarding the biomarkers of IBD, C-reactive protein (CRP) is an effective marker for assessing inflammatory bowel disease because it is appropriate for evaluating treatment efficacy through repeated measurement [4,5].

Another biomarker that has been studied extensively is leucine-rich α2 glycoprotein (LRG), a protein produced in cells such as hepatocytes, neutrophils, and macrophages that is induced by multiple inflammatory cytokines, such as tumor necrosis factor (TNF)-α, IL-22, IL-1β, and IL-6; it is more likely to reflect intestinal inflammation than CRP [6,7]. Fecal calprotectin (FCP) is a protein most abundant in the cytoplasm, granulocytes, and monocytes of intestinal epithelial cells. Assessing this calprotectin protein is essential to observe inflammatory cells infiltrating the intestinal mucosa, injuring the intestinal epithelial cells, and mixing with feces during inflammation [8].

On the other hand, the association between intestinal ultrasound (IUS), a noninvasive, accurate, and well-tolerated tool that provides real-time assessment of inflammatory bowel disease (IBD) activity [9,10], and FCP is a proper screening strategy to identify patients who truly require endoscopy for suspected IBD [11]. This Special Issue of the *Journal of Clinical Medicine* explores inflammatory bowel disease (IBD) and possible clinical diagnosis and treatment, providing a new comprehensive overview of IBD.

Akhmedzyanova et al. investigated the efficacy of telemonitoring in comparison with face-to-face appointments in Russian IBD patients. This study observed that telemonitoring effectively improved clinical, social, and organizational aspects of the Russian healthcare system for IBD patients [12]. Gisbert et al. observed that there are significant variations in the mistakes made when managing patients with IBD in clinical practice. These authors suggest a clear need for the considerable dissemination of clinical practice guidelines among gastroenterologists and the implementation of ongoing training activities supported by scientific societies [13].

In ref. [14], Purnak et al. provide an overview of IBD treatments, modern biologic therapies, and different molecular agents for this disease. These studies focus on improving the symptoms and sustained clinical remission, morphological and functional healing, and providing a better quality of life for these patients. In this sense, different therapeutic approaches have been developed in recent years. Colwill et al., in their review, observed that mirikizumab, a monoclonal antibody directed against the p19 subunit of interleukin (IL)-23, has shown good efficiency and safety for use in ulcerative colitis and Crohn’s disease patients, suggesting that it may be a suitable treatment for elderly patients and those with multiple comorbidities [15].

Regarding physical exercise and gastrointestinal disorders, different studies have investigated the role of exercise in IBD. In this sense, Severo et al. observed in their review that in recent years, new techniques of cellular and molecular biology, and specific gastrointestinal receptors and different hormones, could help us to understand the mechanisms of gastrointestinal changes associated with physical exercise at various intensities, both in experimental and clinical studies [16]. On the other hand, Cagir et al., in their study, described inflammation and oral manifestations during Crohn’s disease (CD). These manifestations include nonspecific and specific lesions that can be overlooked in CD and are sometimes challenging to treat. In this study, 14.2% of CD patients had oral lesions (specific, nonspecific) and 1.2% of CD patients had specific oral lesions affecting their quality of life, inducing pain and weight loss [17].

Bermont et al. investigated the terminal ileitis often identified on computed tomography scans in emergency settings to differentiate CD from other causes of acute terminal ileitis and develop a model for CD diagnosis. These authors identify new predictors of CD amongst patients presenting with acute terminal ileitis through a comprehensive assessment of clinical, laboratory, and imaging characteristics [18]. Ayoub et al. observed a relationship between inflammatory bowel disease (IBD) and pregnancy. The risks of adverse pregnancy outcomes associated with IBD are crucial for effective pregnancy management and support. These authors assess the complications that occur during pregnancy in patients with IBD. Thus, the authors describe that patients with chronic inflammatory bowel diseases can safely become pregnant, provided that they are in remission before and during pregnancy [19].

Pueschel et al. investigate the relationship between inflammatory bowel disease, gastrointestinal function, and the modification of fecal and flatulence odor due to changes in inflammation associated with intestinal microbiota and metabolism. The authors observed the significance of dietary factors and nutrition in managing IBD symptoms, focusing on flatulence and fecal odor [20].

Górecka et al., in their study, investigate the association between inflammatory bowel disease and the complex interplay of immune and proteolytic mechanisms. In this sense, different biomarkers such as neutrophil elastase (NE), which may be released during inflammation, can be assessed. Thus, the authors evaluate the diagnostic and prognostic utility of urinary NE, elafin, an endogenous NE inhibitor, and their ratio in IBD patients. The authors conclude that all analyzed biomarkers—neutrophil elastase, elafin, and the NE/elafin ratio—demonstrated significant potential for diagnosing IBD [21]. Bilican et al. present another method of diagnosing IBD in their study, the intestinal ultrasound (IUS). This diagnostic method is a noninvasive tool used to manage inflammatory bowel disease (IBD), offering real-time, radiation-free assessment of bowel wall thickness, vascularity, and complications. The authors observed that IUS provides a patient-centered, cost-effective imaging option for IBD management, reducing reliance on invasive procedures and radiation exposure while providing real-time insights into disease activity [22].

In conclusion, this Special Issue presents a variety of high-quality studies that contribute to advancing knowledge in the field of IBD associated with diagnostics and treatment.
